# Pharmacological Investigations of N-Substituent Variation in Morphine and Oxymorphone: Opioid Receptor Binding, Signaling and Antinociceptive Activity

**DOI:** 10.1371/journal.pone.0099231

**Published:** 2014-06-11

**Authors:** Tanila Ben Haddou, Szabolcs Béni, Sándor Hosztafi, Davide Malfacini, Girolamo Calo, Helmut Schmidhammer, Mariana Spetea

**Affiliations:** 1 Department of Pharmaceutical Chemistry, Institute of Pharmacy and Center for Molecular Biosciences, University of Innsbruck, Innsbruck, Austria; 2 Department of Pharmaceutical Chemistry, Semmelweis University, Budapest, Hungary; 3 Department of Medical Sciences, Section of Pharmacology and Italian Institute of Neuroscience, University of Ferrara, Ferrara, Italy; Medical School of Hannover, Germany

## Abstract

Morphine and structurally related derivatives are highly effective analgesics, and the mainstay in the medical management of moderate to severe pain. Pharmacological actions of opioid analgesics are primarily mediated through agonism at the µ opioid peptide (MOP) receptor, a G protein-coupled receptor. Position 17 in morphine has been one of the most manipulated sites on the scaffold and intensive research has focused on replacements of the 17-methyl group with other substituents. Structural variations at the N-17 of the morphinan skeleton led to a diversity of molecules appraised as valuable and potential therapeutics and important research probes. Discovery of therapeutically useful morphine-like drugs has also targeted the C-6 hydroxyl group, with oxymorphone as one of the clinically relevant opioid analgesics, where a carbonyl instead of a hydroxyl group is present at position 6. Herein, we describe the effect of N-substituent variation in morphine and oxymorphone on *in vitro* and *in vivo* biological properties and the emerging structure-activity relationships. We show that the presence of a *N*-phenethyl group in position 17 is highly favorable in terms of improved affinity and selectivity at the MOP receptor, potent agonism and antinociceptive efficacy. The *N*-phenethyl derivatives of morphine and oxymorphone were very potent in stimulating G protein coupling and intracellular calcium release through the MOP receptor. *In vivo*, they were highly effective against acute thermal nociception in mice with marked increased antinociceptive potency compared to the lead molecules. It was also demonstrated that a carbonyl group at position 6 is preferable to a hydroxyl function in these *N*-phenethyl derivatives, enhancing MOP receptor affinity and agonist potency *in vitro* and *in vivo*. These results expand the understanding of the impact of different moieties at the morphinan nitrogen on ligand-receptor interaction, molecular mode of action and signaling, and may be instrumental to the development of new opioid therapeutics.

## Introduction

**Figure 1:**
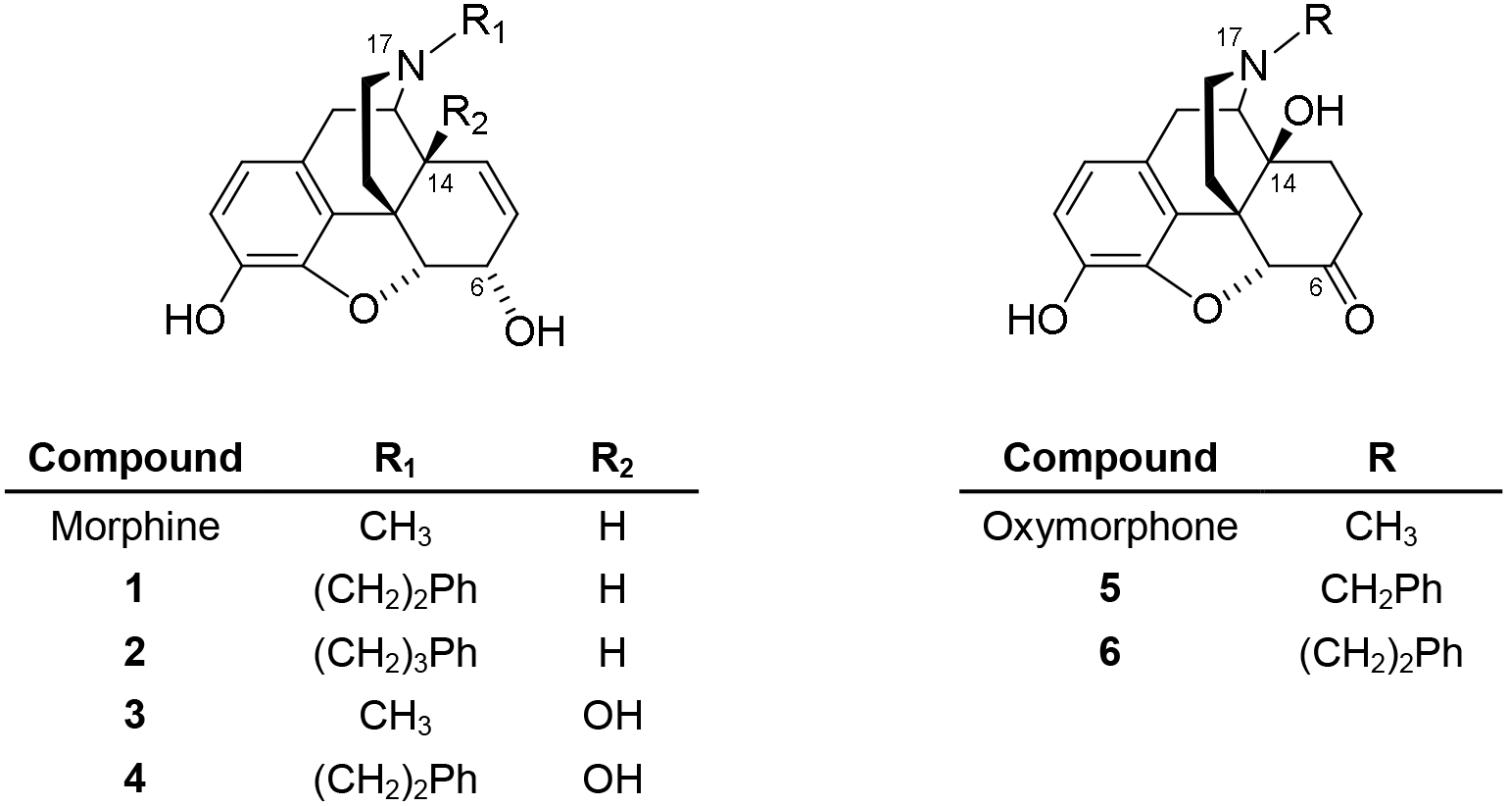
Structures of morphine, oxymorphone and N-substituted morphinans 1–6. Ph, phenyl.

The naturally occurring morphine (Figure 1), the active component of opium, has been used as an analgesic for centuries [1]. Today, effective pain control is still one of the most important therapeutic priorities [2]. Morphine and other structurally related derivatives as well as opioids with distinct structures such as fentanyl have proven to be of the utmost importance as effective analgesics for the treatment of moderate to severe pain. The pharmacological actions of clinically used opioid analgesics are primarily mediated through activation of the µ opioid peptide (MOP) receptor [3], highly expressed in the central and peripheral nervous system and various peripheral tissues. The MOP receptor together with the other members of the opioid receptor class, i.e. δ opioid peptide (DOP) and κ opioid peptide (KOP) receptors, belong to the family of G protein-coupled receptors (GPCRs), and their crystal structures are now available [4]–[6]. While extremely efficacious as pain relievers, opioid analgesics produce an array of side effects that can limit their clinical usefulness, including constipation, nausea, vomiting, and respiratory depression. Long-term treatment with opioids is also associated with development of tolerance to their analgesic effects, physical dependence and addiction [7].

Since the structure elucidation of morphine ninety years ago, its skeleton and its conversion to new analogues was intensively investigated. Consequently, the morphinan skeleton has been the basis of successful drug development, and several opioid drugs are available for patient use or are employed as research probes to examine opioid mechanisms at cellular and molecular levels [3], [8]–[13]. Extensive work in the field led to innovative molecules with new substitution patterns and more favorable pharmacological features, powerful analgesia and less undesirable effects. Established and generally accepted structure-activity relationship (SAR) models have assigned a significant role to the N-substituent in position 17 on the morphinan skeleton in defining the pharmacological behavior. Nalorphine, the *N*-allyl substituted analogue of morphine, was one of the first compounds to be recognized as an opioid antagonist, reversing the analgesic and respiratory depressant actions of morphine [13], [14]. Further studies described that nalorphine alone can induce an antinociceptive effect, which was almost comparable to that of morphine [15], [16], thus defining nalorphine as a partial agonist. Earlier reports on large series of differently N-substituted derivatives of morphine provided exciting outcomes. Exchanging the methyl group at the nitrogen of morphine by other alkyl groups reduces or abolishes analgesic activity [17]. *N*-Phenacyl-, *N*-phenoxyethyl-, and *N*-benzylnormorphine have less than one-tenth of the analgesic potency of morphine [17]. In contrast, it was described that *N*-phenethyl substitution resulted in a 6- to 10-fold higher analgesic potency compared to morphine in rodents, while *N*-cyclohexylethylnormorphine was only one-third as effective [17]. Another targeted site on the morphine skeleton is the C-14 position, where introduction of a hydroxyl group induces an analgesic action of moderate strength [18]. Numerous highly potent morphine-like compounds are known, one of them being oxymorphone, a MOP agonist (Figure 1). Oxymorphone is used not only clinically [19], but also as a valuable scaffold for the development of new ligands interacting with the MOP receptor [9], [11], [20]. A representative example of the complex role played by the morphinan nitrogen in determining the pharmacological properties includes N-substituted derivatives of oxymorphone, ranging from potent agonism i.e. *N*-methyl, *N*-benzyl and *N*-phenethyl, to partial agonism i.e. *N*-dimethylallyl (nalmexone) and *N*-cyclobutylmethyl (nalbuphone), to pure and potent antagonism i.e. *N*-allyl (naloxone) and *N*-cyclopropylmethyl (naltrexone). Substitution of the methyl with a phenylethyl group at the nitrogen in oxymorphone produces a 12-fold increase in analgesic potency [21]. Naloxone and naltrexone, the *N*-allyl- and *N*-cyclopropylmethyl analogues of oxymorphone, respectively, are two opioid antagonists clinically used for the treatment of opioid induced respiratory depression and overdose, with naltrexone being also used for the management of opioid and alcohol dependence [22], [23]. In both morphine and oxymorphone series, it has been reported that the 14-hydroxy group can influence the morphine-like pharmacological profile for varying N-substituents [24], [25]. In the class of agonists, the C14-hydroxyl appears to slightly reduce intrinsic *in vitro* potency, while increasing *in vivo* potency. In partial agonists, the 14-hydroxyl group considerably contributes in decreasing efficacy.

The present study was undertaken to characterize and to compare the effect of N-substituent variation in morphine and oxymorphone on *in vitro* (binding and functional activity) and *in vivo* (nociception) pharmacological properties. SAR studies were performed on a series comprising of four derivatives of morphine (**1–4**) and two derivatives of oxymorphone (**5** and **6**) (Figure 1). Although the synthesis of compounds **1** [26], [27] and **3** [18] has been reported about fifty years ago, and derivative **4** was prepared twenty years ago [28], there is only spare data on their biological activities, with binding affinities and selectivities at MOP, DOP and KOP receptors not yet reported. Herein, we also describe the synthesis and biological characterization of a new N-substituted derivative of morphine, *N*-phenylpropylnormorphine (**2**). In the oxymorphone series, the *N*-phenethyl substituted derivative **6** was already prepared in the 1960s [29] and known as a potent opioid analgesic [21], while *N*-benzylnoroxymorphone (**5**) was synthesized and *in vitro* binding and *in vivo* behavioral studies were first reported by May et al. [30]. To our knowledge, there are no *in vitro* functional activity data at opioid receptors available on any of the investigated morphine and oxymorphone derivatives. We have evaluated the ability of these compounds to stimulate G protein coupling (guanosine 5′-*O*-(3-[^35^S]thio)triphosphate, [^35^S]GTPγS, functional assay) in membranes of cells expressing the human recombinant opioid receptors. Moreover, in cells co-expressing opioid receptors and chimeric G proteins that force the receptor to signal through the calcium pathway, these opioid ligands were examined for their capability to promote calcium mobilization. Furthermore, *in vivo* efficacy in mice against acute thermal nociception (hot-plate and tail-flick tests) was examined and compared to antinociceptive potencies of the lead molecules, morphine and oxymorphone. These investigations provide valuable insights on SAR in the morphinan class of opioids, by broadening our current understanding of the impact of different moieties at the morphinan nitrogen on ligand-receptor interaction, signaling and the link between analgesic efficacy and the molecular mode of action.

## Materials and Methods

### Ethics Statement

All animal studies were conducted in accordance with ethical guidelines and animal welfare standards according to Austrian regulations for animal research, and were approved by the Committee of Animal Care of the Austrian Federal Ministry of Science and Research. Every effort was made to minimize both the animal suffering and the number of animals used.

### Compounds and Reagents

Opioid radioligands, [^3^H][D-Ala^2^,Me-Phe^4^,Gly-ol^5^]enkephalin ([^3^H]DAMGO), [^3^H]5α,7α,8β-(−)*N*-methyl-*N-*[7-(1-pyrrolidinyl)-1-oxaspiro(4,5)dec-8-yl]benzeneacetamide ([^3^H]U69,593) and [^35^S]GTPγS were purchased from PerkinElmer (Boston, USA). [^3^H][Ile^5,6^]deltorphin II was obtained from the Institute of Isotopes Co. Ltd. (Budapest, Hungary). DAMGO, [D-Pen^2^,D-Pen^5^]enkephalin (DPDPE), naloxone, tris(hydroxymethyl)aminomethane (Tris), 2-[4-(2-hydroxyethyl)piperazin-1-yl]ethanesulfonic acid (HEPES), unlabeled GTPγS, guanosine diphosphate (GDP) were obtained from Sigma-Aldrich Chemicals (St. Louis, MO, USA). All cell culture media and supplements were from Sigma-Aldrich Chemicals (St. Louis, MO, USA) and Invitrogen (Paisley, UK). Morphine was obtained from Gatt-Koller GmbH (Innsbruck, Austria). All other chemicals were obtained from standard commercial sources.

The synthesis of *N*-phenethylnormorphine (**1**) was performed from normorphine by alkylation with 2-phenylethyl bromide according to Clark et al. [26] using *N,N*-dimethylformamide (DMF) instead of ethanol as solvent, which provided higher yields. Similarly, *N*-phenylpropylnormorphine (**2**) was synthesized from normorphine using 3-phenylpropyl bromide as alkylating agent. Sodium borohydride reduction of 14-hydroxymorphinone in ethanol yielded 14-hydroxymorphine (**3**) as previously described [18]. *N*-Phenethyl-14-hydroxynormorphine (**4**) was prepared in several steps from *N*-phenethylnorthebaine as earlier described [28]. *N*-Benzylnoroxymorphone (**5**) [31] and *N*-phenethylnoroxymorphone (**6**) [21] were synthesized by a new route via noroxymorphone ethylene ketal. For further details see Chemistry S1.

### In vitro Assays

#### Radioligand binding assays

Membranes were prepared from Sprague-Dawley rat or guinea pig brains as previously described [32]. All binding experiments were performed in 50 mM Tris-HCl buffer (pH 7.4) in a final volume of 1 ml containing 300–500 µg protein [32]. Rat brain membranes were incubated either with [^3^H]DAMGO (1 nM, 45 min, 35°C) or [^3^H][Ile^5,6^]deltorphin II (0.5 nM, 45 min, 35°C). Guinea pig brain membranes were incubated with [^3^H]U69,593 (1 nM, 30 min, 30°C). Nonspecific binding was determined in the presence of 10 µM naloxone. Reactions were terminated by rapid filtration using a Brandel Cell Harvester (Brandel Inc., Gaithersburg, MD) and Whatman GF/B glass fiber filters pre-soaked in 0.1% polyethylenimine for 1 h at 4°C for [^3^H]U69,593, or type GF/C for [^3^H]DAMGO and [^3^H][Ile^5,6^]deltorphin II. Filters were washed three times with 5 ml of ice-cold 50 mM Tris-HCl buffer (pH 7.4) and bound radioactivity was measured by liquid scintillation counting. All experiments were performed in duplicate and repeated at least three times. Protein concentration was determined by the Bradford method using bovine serum albumin as the standard [33].

#### [^35^S]GTPγS functional assays

Chinese hamster ovary (CHO) cells expressing recombinant human MOP, DOP or KOP receptors (CHO_hMOP_, CHO_hDOP_ and CHO_hKOP_ cell lines) were maintained in Dulbecco’s modified Eagle’s medium (DMEM) and Ham F-12 medium supplemented with fetal bovine serum (FBS, 10%), penicillin/streptomycin (0.1%), L-glutamine (2 mM) and geneticin (400 µg/ml) [34]. Cell cultures were maintained at 37°C in 5% CO_2_ humidified air. Membranes were prepared in buffer A (20 mM HEPES, 10 mM MgCl_2_ and 100 mM NaCl, pH 7.4) as described [35]. Cell membranes (5 µg) were incubated with 0.05 nM [^35^S]GTPγS, 10 µM GDP and test compounds for 60 min at 25°C, in a total volume of 1 ml. Nonspecific binding was determined using 10 µM GTPγS, and the basal binding was determined in the absence of test ligand. Samples were filtered over Whatman GF/B glass fiber filters and counted as described for binding assays. All experiments were performed in triplicate and repeated at least three times.

#### Calcium mobilization assays

CHO_hMOP_ and CHO_hKOP_ stably expressing the C-terminally modified Gα_qi5_ protein, and CHO_hDOP_ stably expressing the C-terminally modified Gα_qG66Di5_ protein were grown in DMEM/Ham F-12 medium supplemented with FBS (10%), penicillin (100 IU/ml), streptomycin (100 mg/ml), L-glutamine (2 mM), geneticin (200 µg/ml) and hygromycin B (100 µg/ml). Cell cultures kept at 37°C in 5% CO_2_ in humidified air were used in the calcium mobilization assays performed as previously described [36]. Cells were seeded at a density of 50,000 cells per well into 96-well black, clear-bottom plates. After 24 h, the cells were loaded with medium supplemented with 2.5 mM probenecid, 3 µM of the calcium sensitive fluorescent dye Fluo-4 AM and 0.01% pluronic acid, for 30 min at 37°C. The loading solution was replaced by Hank’s Balanced Salt Solution (HBSS) supplemented with 20 mM HEPES, 2.5 mM probenecid and 500 µM Brilliant Black, for 10 min at 37°C. After placing both plates (cell culture and compound plate) into the FlexStation II (Molecular Device, Union City, CA), fluorescence changes were recorded. All experiments were performed in duplicate and repeated at least three times.

### In vivo Testing

#### Animals

Sprague-Dawley rat and guinea pig brains used in *in* *vitro* assays were obtained from the Institut für Labortierkunde und Laborgenetik, Medizinische Universität Wien (Himberg, Austria). Male CD1 mice (25–30 g) were used in *in* *vivo* studies. Mice were housed in groups of five and were kept in a temperature-regulated environment under a controlled 12 h light/dark cycle with free access to food and water at all times except during testing.

#### Drug administration

Vehicle or solutions of test compounds prepared in sterile physiological saline (0.9%) were administered subcutaneously (s.c.) to mice in a volume of 10 µl per 1 g body weight. At least three doses were tested, and 5–6 animals per dose were used. The dose ranges for the investigated opioids were: morphine (1.25–5 mg/kg), oxymorphone (0.2–1 mg/kg), and compounds **1** (0.05–0.5 mg/kg), **4** (0.5–5 mg/kg), and **6** (0.1–0.5 mg/kg).

#### Nociceptive assessments

The hot-plate test was performed as described [37]. Each mouse was placed on a UB 35100 hot/cold plate (Ugo Basile s.r.l., Varese, Italy) kept at 55°C, and the occurrence of a nociceptive response (licking or shaking a paw, jumping) was observed. To confine the mice to a certain observation area, a colourless plastic cylinder of 20 cm diameter was placed on the hot plate. In order to avoid possible tissue injury, a cut-off time of 12 s was used. The tail-flick test was performed using an UB 37360 Ugo Basile analgesiometer (Ugo Basile s.r.l., Varese, Italy) as previously described [37]. The reaction time required by the mouse to remove its tail due to the radiant heat was measured and defined as the tail-flick latency. A cut-off time of 10 s was used in order to minimize tissue damage.

Hot-plate and tail-flick latencies were measured before (basal latency, BL) and 30, 60 and 120 min after drug or vehicle s.c. administration (test latency, TL). For establishing the dose-response effect, the antinociceptive response was expressed as percent of Maximum Possible Effect (%MPE) = [(TL – BL)/(cut-off time – BL)]×100 for each dose tested.

### Data Analysis

Binding and functional data were analyzed with the GraphPad Prism software (GraphPad Software Inc., San Diego, CA). Concentration-response curves were constructed and inhibition constant (K_i_, nM), agonist potency (EC_50_, nM) and efficacy (E_max_, as percentage of maximum stimulation with respect to the standard opioid agonists, DAMGO (MOP), DPDPE (DOP) and U69,593 (KOP)) were calculated using nonlinear curve fitting analysis. Data are represented as the mean ± SEM. For *in* *vivo* assays, the effective dose ED_50_ and 95% confidence limits (95% CL) were calculated using the method of Litchfield and Wilcoxon [38].

## Results and Discussion

Opioid receptor binding affinities and selectivities of the four derivatives of morphine (**1–4**) and two derivatives of oxymorphone (**5** and **6**) were determined by *in* *vitro* competition binding assays using membranes from rat brain (MOP and DOP opioid receptors) or guinea pig brain (KOP opioid receptors) [32]. Receptor type-specific radioligands were used i.e. [^3^H]DAMGO (MOP), [^3^H][Ile^5,6^]deltorphin II (DOP) and [^3^H]U69,593 (KOP). Binding affinities expressed as K_i_ values and selectivity ratios are listed in Table 1. For comparison purposes, opioid binding affinities of morphine and oxymorphone are included in Table 1.

**Table 1:** Opioid receptor binding affinities and selectivities at MOP, DOP and KOP receptors.

	K_i_ (nM)			Selectivity ratios	
	MOP	DOP	KOP	DOP/MOP	KOP/MOP
Morphine	6.55±0.74	217±19	113±9	33	17
Oxymorphone	0.97±0.05	80.5±5.5	61.6±1.2	83	51
**1**	0.93±0.14	37.0±5.5	107±18	40	115
**2**	79.5±1.1	869±171	565±24	11	7
**3**	16.4±1.1	1,081±271	789±77	66	48
**4**	4.60±0.01	163±17	513±66	35	112
**5**	359±31	1,078±35	75.0±8.0	3	0.2
**6**	0.54±0.03	12.8±0.2	84.2±7.2	24	156

Binding assays were performed with membranes from rat brain (MOP and DOP receptors) and guinea pig brain (KOP receptors).

Values represent the mean ± SEM of at least three experiments each performed in duplicate.

In the series of morphine derivatives, the *N*-phenethyl substituted **1** displayed the highest MOP receptor affinity (K_i_ = 0.93 nM), being 7-fold greater than the affinity of morphine. The other *N*-phenethyl substituted analogue **4** with a 14-hydroxyl group also showed high MOP receptor affinity in the low nanomolar range similar to that of morphine (Table 1). A lower binding affinity to the MOP receptor was exhibited by the new *N*-phenylpropylnormorphine (**2**) and 14-hydroxymorphine (**3**). Generally, all morphine derivatives **1–4** had one to two orders of magnitude decreased affinities at DOP and KOP receptors. Concerning MOP selectivity, with the exception of the new *N*-phenylpropyl substituted **2**, the other three derivatives had comparable selectivity vs. DOP, and increased selectivity vs. KOP receptors. The presence of a hydroxyl group at position 14 in *N*-phenethyl-14-hydroxynormorphine (**4**) resulted in about 5-fold lower affinity at the MOP receptor paralleled by decreased interaction with DOP and KOP receptors as compared to its 14-unsubtituted counterpart **1**, although leaving MOP selectivity unchanged. Furthermore, replacement of the *N*-phenethyl substituent in **1** with a *N*-phenylpropyl group (**2**) largely reduces both binding affinity and selectivity at the MOP receptor (Table 1). Exchanging the 17-methyl group in 14-hydroxymorphine (**3**) with a phenethyl moiety (**4**) increases binding affinity at the MOP receptor by about 4-fold, with a slight decrease in DOP/MOP and an increase in KOP/MOP selectivity ratios.

Compared to the parent molecule oxymorphone, the *N*-phenethyl substituted analogue **6** exhibited almost 2-fold higher affinity at the MOP receptor and better MOP vs. KOP receptor selectivity. On the contrary, the other derivative **5** showed much reduced interaction with MOP receptors, and very low MOP selectivity (Table 1), indicating that a benzyl group at N-17 is not favorable for both MOP affinity and selectivity. Our present observations on the low binding profile at the MOP receptor of the *N*-benzyl substituted derivative of oxymorphone (**5**) agree with previously reported binding data (K_i_ values of 138 nM for MOP, 529 nM for DOP, and 134 nM for KOP) [30]. *N*-Phenethylnoroxymorphone (**6**) displayed about 9-fold increased MOP receptor affinity and similar DOP/MOP and KOP/MOP selectivity ratios compared to its analogue in the morphine series, *N*-phenethyl-14-hydroxynormorphine (**4**). Similar observations are made when comparing oxymorphone with its 14-hydroxy analogue **3** (Table 1).

**Figure 2:**
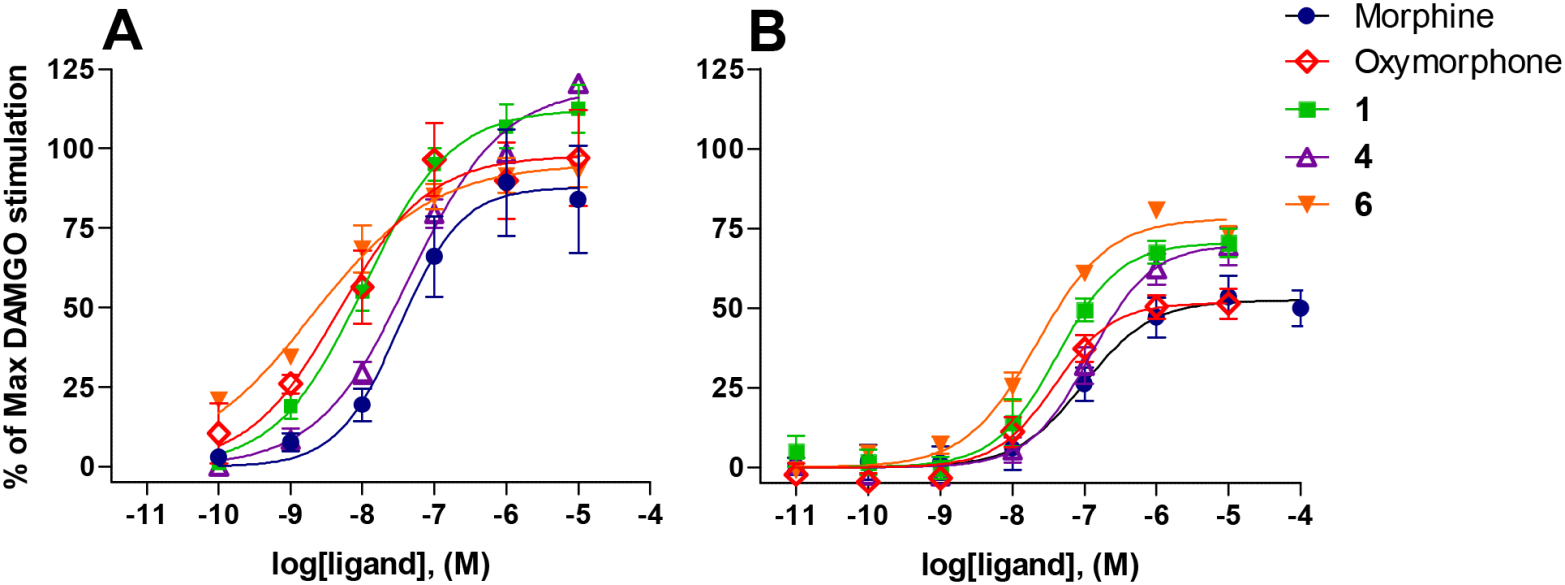
*In vitro* agonist activities at the MOP receptor of morphine, oxymorphone and *N*-methylmorphinans 1, 4 and 6. Concentration-response curves in (A) [^35^S]GTPγS functional assay with membranes from CHO expressing human MOP receptor and (B) calcium mobilization experiments performed with CHO cells co-expressing the human MOP receptor and the Gα_qi5_ protein. Activity is calculated as percentage of maximal stimulation produced by DAMGO. Data are shown as the mean ± SEM (*n*≥3).

Among the investigated *N*-phenethyl substituted compounds, the two morphine derivatives **1** and **4** and the oxymorphone analogue **6**, showing high binding affinity and selectivity at the MOP receptor (Table 1), were selected for further *in* *vitro* and *in* *vivo* studies. The other derivatives (**2**, **3** and **5**), due to their reduced interaction with the MOP receptor together with much lower binding affinities to this receptor than the parent molecules morphine or oxymorphone (Table 1), where not considered for additional investigations. First, compounds **1**, **4** and **6** were examined for agonist potencies and efficacies *in* *vitro.* In this study it was of utmost relevance to assess initially MOP receptor-mediated G protein activation by these opioid agonists. The used functional approaches assessed stimulation of [^35^S]GTPγS binding and intracellular calcium mobilization. The effect of the investigated compounds was compared to that of the parent molecules morphine and oxymorphone, and to the profile of the standard opioid agonists, DAMGO (MOP), DPDPE (DOP) and U69,593 (KOP). Potencies as EC_50_ values and efficacies as maximum response (E_max_) to the reference opioid agonists are presented in Table 2. Opioid receptor-mediated G protein signaling was evaluated using a [^35^S]GTPγS binding assay in membranes from CHO cells stably expressing either the human MOP, DOP or KOP receptors [35]. In CHO_hMOP_ cell membranes, all three compounds **1**, **4** and **6** produced concentration-dependent increase in [^35^S]GTPγS binding (Figure 2A). Oxymorphone derivative **6** was the most potent MOP agonist with an EC_50_ value of 2.63 nM, roughly equivalent to that of oxymorphone, while also showing similar efficacies. This *N*-phenethyl substituted **6** also proved to be more potent than DAMGO (EC_50_ = 19.3 nM) in stimulating G protein signaling. High agonist potency was also depicted by the morphine derivative **1**, being about 3-fold more potent as MOP agonist than morphine, and 2-fold than DAMGO. In contrast, the *N*-phenethyl-14-hydroxynormorphine (**4**) exhibited the lowest potency having an EC_50_ value similar to morphine (Table 2). The rank order of agonist potencies to promote MOP receptor mediated-G protein coupling correlates well with binding affinities at the MOP receptor observed in the radioligand binding studies (Table 1).

**Table 2:** *In vitro* agonist potency and efficacy.

Compound	[^35^S]GTPγS functional assay^a^						Calcium mobilization assay^b^					
	MOP		DOP		KOP		MOP		DOP		KOP	
	EC_50_ (nM)	E_max_ (%)^c^	EC_50_ (nM)	E_max_ (%)^c^	EC_50_ (nM)	E_max_ (%)^c^	EC_50_ (nM)	E_max_ (%)^c^	EC_50_ (nM)	E_max_ (%)^c^	EC_50_ (nM)	E_max_ (%)^c^
Morphine	34.4±5.1	89±17	668±65	109±14	710±23	76.1±2.0	140±31	55±3	inactive^d^		2,185±451	51±2
Oxymorphone	4.38±0.76	98±11	259±33	87±40	463±116	48±11	44.3±9.7	52±5	inactive^d^		inactive^d^	
**1**	10.3±0.9	113±8	712±86	138±17	1,049±29	19±2	48.8±14.0	70±4	crc incomplete^e^		inactive^d^	
**4**	46.3±7.1	119±3	1,247±356	125±15	ND^f^		124±20	70±7	inactive^d^		inactive^d^	
**6**	2.63±1.06	97±3	131±60	101±9	225±74	7.5±0.01	23.4±4.7	78±2	crc incomplete^e^		inactive^d^	

a Membranes from CHO cells stably transfected with human MOP, DOP or KOP receptors were used.

b CHO cells co-expressing chimeric G proteins and recombinant human MOP, DOP or KOP receptors.

c E_max_ is expressed in percentage relative to maximal stimulation produced by DAMGO (MOP), DPDPE (DOP) or U69,593 (KOP).

d Inactive up to 10 µM.

e crc, concentration response curve.

f ND, not determined due to very low binding affinity at the KOP receptor.

Values represent the mean ± SEM of at least three experiments each performed in duplicate or triplicate.

By comparing the agonist potency at the hMOP receptor expressing CHO cells, potencies of derivatives **1**, **4** and **6**, morphine and oxymorphone to stimulate [^35^S]GTPγS binding were decreased considerably in hDOP (EC_50_ = 3.0 nM for DPDPE) and hKOP receptors (EC_50_ = 42.7 nM for U69,593) expressing cells (Table 2). While in CHO_hDOR_ cell membranes, they showed high efficacies, much reduced to no stimulation was measured at the KOP receptor. Due to very low binding affinity at the KOP receptor (K_i_ = 513 nM), we did not investigate the activity at the KOP receptor of compound **4** in the [^35^S]GTPγS binding.

In this study, we have also examined the potency and efficacy of derivatives **1**, **4** and **6** to evoke changes in intracellular calcium concentration using a whole cell fluorescence-based assay [36]. In CHO_hMOP_ cells stably expressing the Gα_qi5_ chimeric protein, all compounds produced a concentration-dependent stimulation of calcium release (Figure 2B). It is notable that the rank order of EC_50_ values correlated well with the EC_50_ values obtained in [^35^S]GTPγS binding assays (Table 2), with *N*-phenethylnoroxymorphone (**6**) showing the highest potency. Compared to DAMGO (EC_50_ = 42.7 nM), compound **6** was about 2-fold more potent. Among the two morphine derivatives, *N*-phenethylnormorphine (**1**) was about 3-fold more potent than morphine and equipotent to DAMGO, and about 3-fold more active than its 14-hydroxy analogue **4** in evoking calcium mobilization (Table 2). In CHO_hDOP_ cells expressing the Gα_qG66Di5_ chimeric protein, and in CHO_hKOP_ cells expressing the Gα_qi5_ chimeric protein, the investigated derivatives stimulated calcium release with considerably lower potencies or were even found inactive, which is in line with the findings from [^35^S]GTPγS functional assays (Table 2).

**Figure 3:**
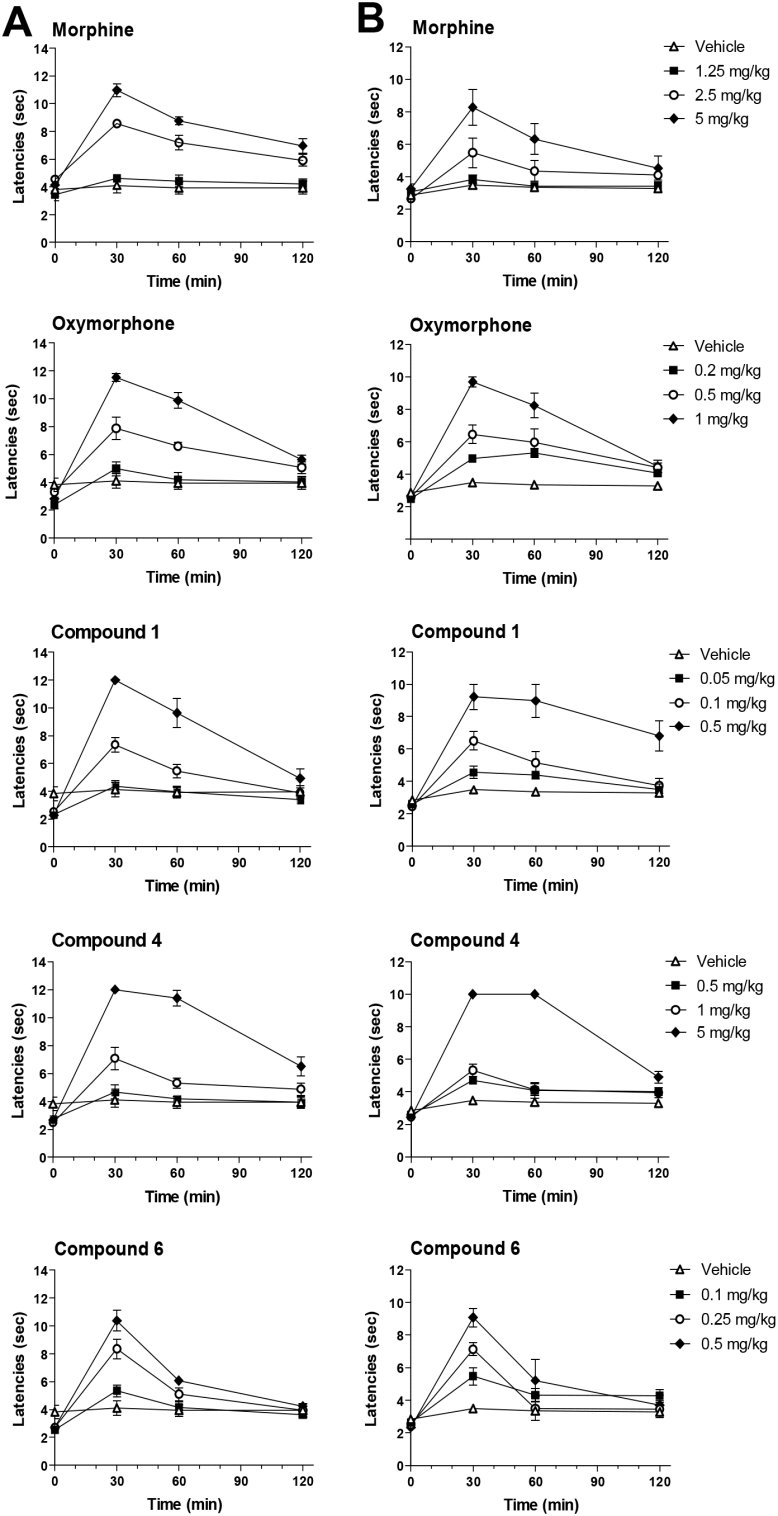
Time-course of antinociceptive effects produced by morphine, oxymorphone and *N*-methylmorphinans 1, 4 and 6. The effect of morphine (1.25–5 mg/kg), oxymorphone (0.2–1 mg/kg), and compounds **1** (0.05–0.5 mg/kg), **4** (0.5–5 mg/kg), and **6** (0.1–0.5 mg/kg) in the hot-plate test (A, left panel) and in the tail-flick test (B, right panel). Hot-plate and tail-flick latencies (in seconds) were determined in mice before (0 min) and after s.c. drug administration (30, 60 and 120 min). Data are shown as the mean ± SEM (*n* = 5–6 mice per group).

**Figure 4:**
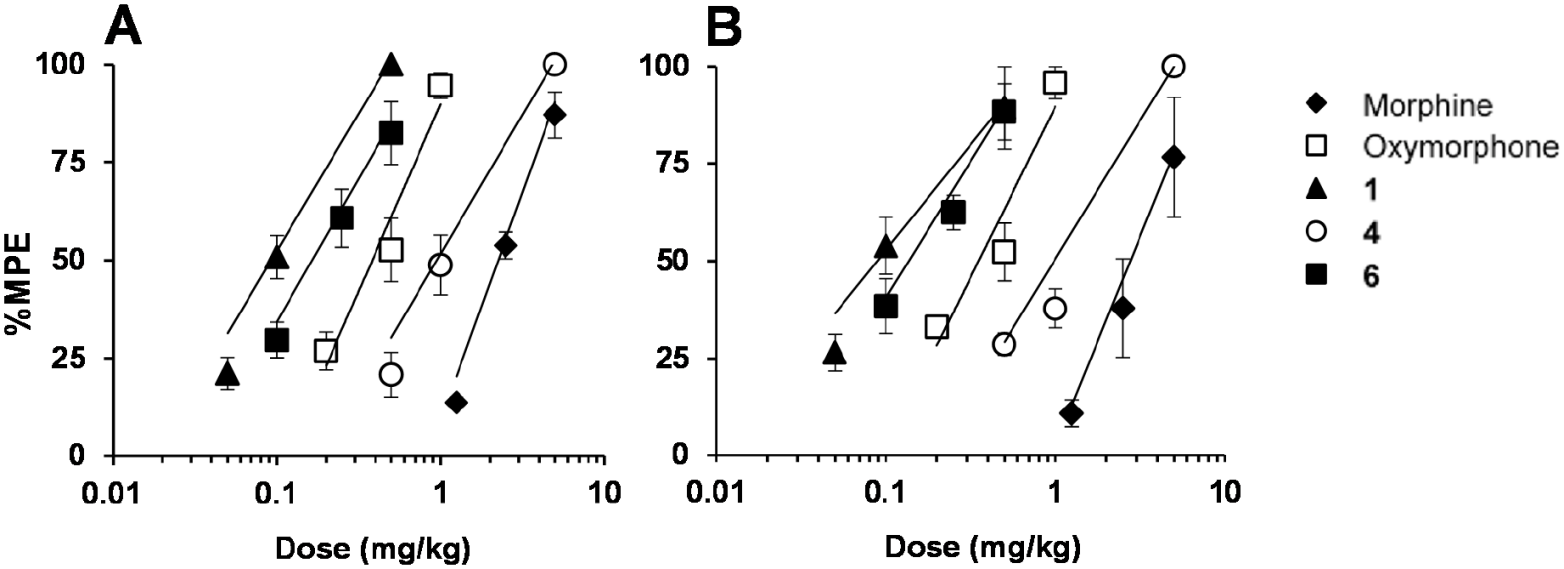
Dose-dependent antinociceptive effects produced by morphine, oxymorphone and *N*-methylmorphinans 1, 4 and 6. (A) Hot-plate test. (B) Tail-flick test. Hot-plate and tail-flick latencies (as %MPE) are shown at 30 min (peak of action) after s.c. drug administration to mice. Data are shown as the mean ± SEM (*n* = 5–6 mice per group).

The findings from our *in* *vitro* studies including binding affinity and potency at the MOP receptor together with earlier reports on the analgesic effects of compounds **1** [17] and **6** [21] and preliminary experiments were used to establish the appropriate dose range for *in* *vivo* investigations. Antinociceptive properties of morphine derivatives **1** and **4**, and oxymorphone analogue **6** were assessed in mice after s.c. administration using two nociceptive tests, hot-plate and tail-flick [37]. All three MOP agonists produced time- and dose-dependent effects in both nociceptive assays (Figures 3 and 4) with compounds **1** and **6** being the most effective against acute thermal nociception. The peak antinociception occurred generally 30 min after drug s.c. administration (Figure 3). Antinociceptive potencies expressed as ED_50_ values with 95% confidence limits are listed in Table 3, and were compared with those of the reference opioids drugs, morphine and oxymorphone. In agreement with the earlier observations of Winter et al. [17], morphine derivative **1** was also shown in our study to be a more potent antinociceptive than morphine. In the hot-plate and tail-flick tests, it was 22- and 28-fold, respectively, more effective than morphine. First data on the antinociceptive effect of *N*-phenethyl-14-hydroxynormorphine (**4**) are described herein, revealing this MOP agonist as a potent antinociceptive agent with a 2- to 3-fold increased potency than morphine. Compound **6**, the *N*-phenethyl analogue of oxymorphone, was found to be highly active with about 2-fold higher potency than oxymorphone, and comparable potency to **1**. It was up to 8-fold more potent than its 6-hydroxy counterpart **4** in inducing an antinociceptive response, indicating a 6-keto substitution to be preferable toward improved analgesic properties. Besides analgesia, MOP agonists are well-known to induce other behavioral changes. While in this study, generally, no major alterations in locomotor activity and no sedative effects were observed at any of the tested doses of compounds **1**, **4** and **6**, representing about 3- to 4-fold the analgesic ED_50_ dose, further investigations will be needed to establish the therapeutic index of these compounds.

**Table 3:** Antinociceptive activities.

	ED_50_ (mg/kg, s.c.) (95% CL)^a^	
	Hot-plate test	Tail-flick test
Morphine	2.43 (1.38–4.27)	3.06 (1.76–5.31)
Oxymorphone	0.38 (0.19–0.78)	0.35 (0.16–0.77)
**1**	0.11 (0.045–0.26)	0.11 (0.027–0.40)
**4**	1.12 (0.46–2.69)	1.14 (0.45–2.87)
**6**	0.18 (0.074–0.46)	0.15 (0.058–0.40)

a Antinociceptive potencies determined 30 min after s.c. drug administration in mice shown as ED_50_ values with 95% confidence limits (95% CL) (*n* = 5–6 mice per group).

## Conclusions

Position 17 in morphine has been one of the most manipulated sites on the scaffold and intensive research has focused on replacements of the 17-methyl group with other substituents. Structural variations at the N-17 of the morphinan skeleton have resulted in a diversity of compounds appraised as valuable and therapeutic agents and important research tools [3], [9], [11], [12]. Furthermore, discovery of therapeutically useful morphine-like drugs has also targeted the C-6 hydroxyl group, with oxymorphone as one example of the clinically relevant opioid analgesics, where a carbonyl instead of a hydroxyl group is present at position 6 [9], [39]. Taken together, in the present study we highlight on the significant outcomes of N-substituent variation in morphine and oxymorphone on *in* *vitro* and *in* *vivo* biological properties and the emerging SAR. The presented data clearly reflect that a *N*-phenethyl moiety in position 17 is highly favorable regarding enhanced affinity and selectivity at the MOP receptor, potent agonism and antinociceptive action. The increased lipophilicity of the *N*-phenethyl derivatives compared to the parent compounds may also contribute to the increased potency. Besides, it was also demonstrated that a carbonyl group at position 6 is preferable to a hydroxyl function in the *N*-phenethyl substituted molecules, augmenting MOP receptor affinity and agonist potency *in* *vitro* and *in* *vivo*. Though morphine derivatives, *N*-phenethylnormorphine (**1**) and *N*-phenethyl-14-hydroxynormorphine (**4**), and the oxymorphone analogue *N*-phenethylnoroxymorphone (**6**) have been developed many years ago, this is the first report on their opioid receptor binding and signaling, and antinociceptive efficacy. This report clarifies the activity of these molecules at the opioid receptors for the first time, serving as a systematic study of understanding their mode of action and the link between agonist-induced G protein signaling events leading to the high analgesic efficacy. Moreover, these results reveal that targeting position 17 is a viable approach toward improving the pharmacological properties, and may be instrumental to the development of new opioids for therapeutic use in the clinic. Considering the interesting functional profile of these MOP agonists and their high efficacy as antinociceptive agents, it is of interest to investigate other intracellular signaling pathways (i.e. interactions with regulatory proteins such as β-arrestins) and their side-effect profile in future studies.

## Supporting Information

Chemistry S1 (DOCX)
