# Supplementary material for: Pharmacological Investigations of N-Substituent Variation in Morphine and Oxymorphone: Opioid Receptor Binding, Signaling and Antinociceptive Activity
Source: PLoS One. 2014 Jun 11;9(6):e99231. doi: 10.1371/journal.pone.0099231 (PMC4053365; doi:10.1371/journal.pone.0099231)
Supplement: Chemistry S1 — (DOCX) [file pone.0099231.s001.docx]

**Supplemental Chemistry S1**

1. **General methods**

All reagents and solvents were purchased from Sigma-Aldrich and were used as received. Melting points were measured with a Büchi apparatus in open capillary tubes. TLC was performed on silica gel 60 F254 (Merck-5719) plates using the following three eluent systems: benzene:methanol 8:2, chloroform:methanol 9:1, chloroform:acetone:diethylamine 5:4:1. The spots were visualized by ultraviolet (UV) light or with Dragendorff reagent. For column chromatography, Kieselgel 60 (particle size 0.063–0.2 mm) was employed with eluent system chloroform:methanol 9:l. High-resolution accurate masses were determined with an Agilent 6230 time-of-flight mass spectrometer. Samples were introduced by the Agilent 1260 Infinity LC system and the mass spectrometer was operated in conjunction with a Jet Stream electrospray ion source in positive ion mode. Reference masses of m/z = 121.050873 and 922.009798 were used to calibrate the mass axis during analysis. Mass spectra were processed using Agilent MassHunter B.02.00 software. Electron ionization mass spectra were obtained with a VG Trio-2 instrument using the EI method (70 eV). NMR spectra were recorded on a 600 MHz Varian DDR NMR spectrometer equipped with a 5 mm inverse-detection gradient (IDPFG) probe at 298 K. Chemical shifts are reported in parts per million (δ) relative to tetramethylsilane (TMS) as internal standard; coupling constants (*J*) are given in Hz.

1. **Synthesis**

**N-Aralkylation**

To a stirred solution of normorphine or noroxymorphone ethylene ketal (2 mmol) in DMF was added sodium hydrogencarbonate (0.5 g, 6 mmol) followed by the addition of the appropriate aralkyl bromide (2.2 mmol; benzyl bromide 0.26 ml or 2-phenylethyl bromide 0.3 ml or 3-phenylpropyl bromide 0.33 ml). The reaction mixture was stirred and heated at 80°C for 16 h. Inorganic salts were filtered off and the filtrate was evaporated to dryness in vacuo. The residue was diluted with 5% ammonia solution and extracted three times with chloroform-isopropanol (3:1, v/v) mixture. The resulting extracts were combined and dried (sodium sulfate). The crude products were purified by recrystallization and/or column chromatography.

Noroxymorphone shows low solubility in ethanol and DMF. Therefore, noroxymorphone ethylene ketal has been prepared, which could be readily aralkylated in ethanol or DMF due to good solubility in these solvents. The ketal protecting group was removed easily by acidic hydrolysis to provide the corresponding arylalkylated oxymorphone derivatives 5 and 6.

# **Noroxymorphone ethylene ketal**

A mixture of noroxymorphone (2.0 g), ethylene glycol (3.0 g) and p-toulenesulfonic acid hydrate (2.0 g) in benzene was (30 ml) refluxed and stirred for 30 hours. Water which formed during the ketalization process was removed with a Dean-Stark trap. After cooling, benzene was removed under reduced pressure and the residue was dissolved in water. The resulting solution was made alkaline with 10% ammonia. The precipitate was isolated and recrystallized.

Noroxymorphone Noroxymorphone ethylene ketal

# Yield 74%, m.p.: 309-312°C dec. (methanol-chloroform); ^1^H-NMR (CDCl_3_) δ 6.70-6.67 (m, overlap of H-1,2, 2H) 4.50 (s, H-5; 1H) 4.15-3.80 (m, ketal ethylene protons, 4H); HRMS: C_18_H_21_NO_5_ calculated 332.1492 (MH^+^), found: 332.1504, EI MS: m/e 331 (M^+^ 26%).

***N*-Benzylnoroxymorphone ethylene ketal**

Noroxymorphone ethylene ketal was aralkylated as described above to yield *N*-benzylnoroxymorphone ethylene ketal. The desired compound was purified by column chromatography and isolated as yellow oil (yield 62%).

^1^H-NMR (CDCl_3_) δ 7.4-7.23 (m, Ar-H, 5H) 6.50 (d, *J* = 8.0 Hz, 1H), 6.48 (d, *J* = 8.0 Hz, 1H), 4.34 (s, H-5, 1H) 4.10-3.80 (m, ketal protons, 4H), 3.65 (s, PhCH_2_, 2H); HRMS: C_25_H_27_NO_5_  calculated 422.1962 (MH^+^), found: 422.1954, EI MS: m/e 421 (M^+^, 8%).

# *N*-Phenylethylnoroxymorphone ethylene ketal

Noroxymorphone ethylene ketal was aralkylated as described above to yield *N*-phenylethylnoroxymorphone ethylene ketal.

Yield 37%, m.p.: 76-78°C (ether); ^1^H-NMR (DMSO-d_6_) δ 7.4-7.22 (m, Ar-H, 5H), 6.67 (d, *J* = 7.2 Hz, 1H), 6.57 (d, *J* = 7.3 Hz, 1H), 4.54 (s, H-5, 1H) 4.10-3.80 (m, ketal protons, 4H); HRMS: C_26_H_29_NO_5_  calculated 436.2118 (MH^+^), found: 436.2121, EI MS: m/e 435 (M^+^; 2 %) 344 (M-91; 100%).

# Hydrolysis of N-aralkylated noroxymorphone ethylene ketals

The respective ketal (2 g) was dissolved in 1N HCl (40 ml) and the solution was heated on a steam bath for 2 hours. After basification with 10% ammonia, the product was extracted with a chloroform-isopropanol (3:1, v/v) mixture**.** The resulting extracts were combined and dried (sodium sulfate). The crude products were purified by recrystallization and/or column chromatography to provide pure 5 and 6.

**Normorphine**

Normorphine was prepared by the method of Olofson and Schnur [1]. Diacetylmorphine was *N*-demethylated with vinyl chloroformate and the resulting carbamate was cleaved with HCl gas. The product 3-*O*,6-*O*-diacetylnormorphine was hydrolyzed with 6% HCl to yield normorphine.

**Noroxymorphone**

Noroxymorphone was prepared by the method described in [2]. 3-*O*,14-*O*-diacetyloxymorphone was N-demethylated with 1-chloroethyl chloroformate, and after cleaving of the intermediate carbamate the resulting 3-*O*,14-*O*-diacetylnoroxymorphone hydrochloride was hydrolyzed with 10% HCl to obtain noroxymorphone.

***N*-Phenylethylnormorphine (1)**

Yield 69%; m.p.: 255-57°C (ethanol); ^1^H-NMR (DMSO-d_6_) δ 7.30-7.15 (m, Ar-H, 5H) 6.44 (d, *J* = 8.0 Hz, 1H), 6.33 (d, *J* = 8.0 Hz, 1H) 5.53 (d, *J* = 9.6 Hz, H-7, 1H), 5.24 (d, *J* = 9.6 Hz, H-8, 1H), 4.84 (d, *J* = 6.3 Hz, H-5, 1H), 4.10 (m, H-6, 1H); HRMS: C_24_H_25_NO_3_ calculated 376.1907 (MH^+^), found: 376.1904, EI MS EI: m/e 284 (M-91; 100%).

***N*-Phenylpropylnormorphine** (**2**)

Normorphine **2**

1. 3-Phenylpropyl bromide, NaHCO_3_, DMF, 80 °C, 20 h.

Yield 77%; m.p.: 187-88°C (ethanol); ^1^H-NMR (DMSO-d_6_) δ 7.30-7.15 (m, Ar-H, 5H) 6.61 (d, *J* = 8.1 Hz, 1H), 6.45 (d, *J* = 8.1 Hz, 1H), 5.62 (d, *J* = 9.5 Hz, H-7, 1H), 5.23 (d, *J* = 9.6 Hz, H-8, 1H), 4.84 (d, *J* = 5.8 Hz, H-5, 1H), 4.17 (br s, H-6, 1H); HRMS: C_25_H_27_NO_3_ calculated 390.2064 (MH^+^), found: 390.2086, EI MS EI: m/e 389 (M+, 14%), 284 (100%).

***N*-Benzylnoroxymorphone (5)**

# Yield 63%, m.p.: 235-37°C (ethanol); ^1^H-NMR (DMSO-d_6_) δ 7.37-7.24 (m, Ar-H, 5H) 6.74 (d, *J* = 8.2 Hz, 1H), 6.62 (d, *J* = 8.2 Hz, 1H), 4.70 (s, H-5, 1H); HRMS: C_23_H_23_NO_4_ calculated 378.1700 (MH^+^), found: 378.1703, EI MS: m/e 377 (M^+^, 15%).

***N*-Phenylethylnoroxymorphone (6)**

Yield 57%, m.p.: 85-90°C (ether), HCl-salt 285-88 °C (ethanol); ^1^H-NMR (CDCl_3_) δ 7.35-7.17 (m, Ar-H, 5H), 6.72 (d, *J* = 8.2 Hz, 1H),, 6.58 (d, *J* = 8.2 Hz, 1H), 4.67 (s, H-5, 1H), 3.08 (d, *J* = 18.4 Hz, 1H) 3.00 (td, *J* = 14.5, 5.0 Hz, 1H), 2.93 (d, *J* = 5.0 Hz, 1H); HRMS: C_24_H_25_NO_4_  calculated 392.1856 (MH^+^), found: 392.1844, EI MS: m/e 391 (M^+^; 3%), 300 (M-91, 100%).

1. **References**
2. Olofson RA, Schnur RC (1977) Value of the vinyloxycarbonyl unit in hydroxyl protection: application to the synthesis of nalorphine. Tetrahedron Lett 18: 1571-1574.
3. Olofson RA, Martz JT, Senet JP, Piteau M, Mafroot T (1984) A new reagent for the selective high-yied N-dealkylation of tertiary amines: improved synthesis of naltrexone and nalbuphine. J Org Chem 49: 2081-2082.
